# Prediction of upcoming urinary tract infection after intracerebral hemorrhage: a machine learning approach based on statistics collected at multiple time points

**DOI:** 10.3389/fneur.2023.1223680

**Published:** 2023-09-14

**Authors:** Yanjie Zhao, Chaoyue Chen, Zhouyang Huang, Haoxiang Wang, Xin Tie, Jinhao Yang, Wenyao Cui, Jianguo Xu

**Affiliations:** ^1^Department of Neurosurgery, West China Hospital, Sichuan University, Chengdu, China; ^2^Department of Critical Care Medicine, West China Hospital, Sichuan University, Chengdu, China

**Keywords:** urinary tract infection, intracerebral hemorrhage, stroke, critical care, machine learning

## Abstract

**Purpose:**

Accurate prediction of urinary tract infection (UTI) following intracerebral hemorrhage (ICH) can significantly facilitate both timely medical interventions and therapeutic decisions in neurocritical care. Our study aimed to propose a machine learning method to predict an upcoming UTI by using multi-time-point statistics.

**Methods:**

A total of 110 patients were identified from a neuro-intensive care unit in this research. Laboratory test results at two time points were chosen: Lab 1 collected at the time of admission and Lab 2 collected at the time of 48 h after admission. Univariate analysis was performed to investigate if there were statistical differences between the UTI group and the non-UTI group. Machine learning models were built with various combinations of selected features and evaluated with accuracy (ACC), sensitivity, specificity, and area under the curve (AUC) values.

**Results:**

Corticosteroid usage (*p* < 0.001) and daily urinary volume (*p* < 0.001) were statistically significant risk factors for UTI. Moreover, there were statistical differences in laboratory test results between the UTI group and the non-UTI group at the two time points, as suggested by the univariate analysis. Among the machine learning models, the one incorporating clinical information and the rate of change in laboratory parameters outperformed the others. This model achieved ACC = 0.773, sensitivity = 0.785, specificity = 0.762, and AUC = 0.868 during training and 0.682, 0.685, 0.673, and 0.751 in the model test, respectively.

**Conclusion:**

The combination of clinical information and multi-time-point laboratory data can effectively predict upcoming UTIs after ICH in neurocritical care.

## 1. Introduction

Manifesting as bacteremia, sepsis, and acute renal failure, urinary tract infection (UTI) is a significant complication linked to unfavorable prognostic outcomes in intracerebral hemorrhage (ICH) patients (1–3). It can result in readmission with poor clinical outcomes, with a mortality rate of 29% within 1 year of onset. The occurrence rate of UTI following ICH is from 15.1% to 26.1%, as reported (4, 5). Therefore, predicting UTI after ICH is essential to facilitate timely medical interventions and precise therapeutic decisions.

Previous studies have attempted to predict UTI after stroke with various methods. A significant number of published studies have demonstrated the prognostic value of patient clinical information in predicting UTI during hospitalization (6–9). Meanwhile, as major advances in immunodepression after stroke have been made (10–12), research has also shown an increased interest in identifying significant infectious-related laboratory results associated with upcoming UTIs to promote more precise clinical interventions (13–18). Furthermore, predictive models for patients with high incidence rates were established using multivariable analysis and achieved moderate performance (14, 19–21).

Machine learning is a branch of artificial intelligence that enables computers to learn from data and make predictions or decisions without being explicitly programmed. By analyzing large and complex datasets of health records, imaging, genetics, environmental factors, and other variables, they can provide personalized and preventive recommendations for patients and clinicians, such as optimal treatment plans, lifestyle interventions, and follow-up actions (22–24). Recent developments in machine learning methods have introduced renewed interest in the prediction, management, and prognosis of stroke patients (25–28). As for the complications, a multi-center study suggested that predicting post-stroke UTI risk in immobile patients with an ensembled machine learning model was promising with the highest area under the curve (AUC) of 0.808 and accuracy (ACC) of 0.703 (29), whereas, despite their contributions, a major limitation that persists in these studies is their exclusive dependence on clinical and/or laboratory parameters obtained only at the time of patient admission. In real clinical settings, patients receive active and systematic treatment, which may influence the progression of the disease. As such, the importance of monitoring variation in laboratory findings has been highlighted as these alterations may serve as indicators of disease progression and the possible emergence of UTI (30). Therefore, a predictive model that incorporates data from multiple time points would offer greater clinical relevance in identifying patients who are at a higher risk for complications.

Thus, this study aims to (1) propose a machine learning model to identify patients who are at an increased risk of developing UTI after ICH at an early stage and (2) investigate if the predictive performance of the model will be improved if multi-time-point information is used.

## 2. Methods

### 2.1. Study population

**Figure 1:**
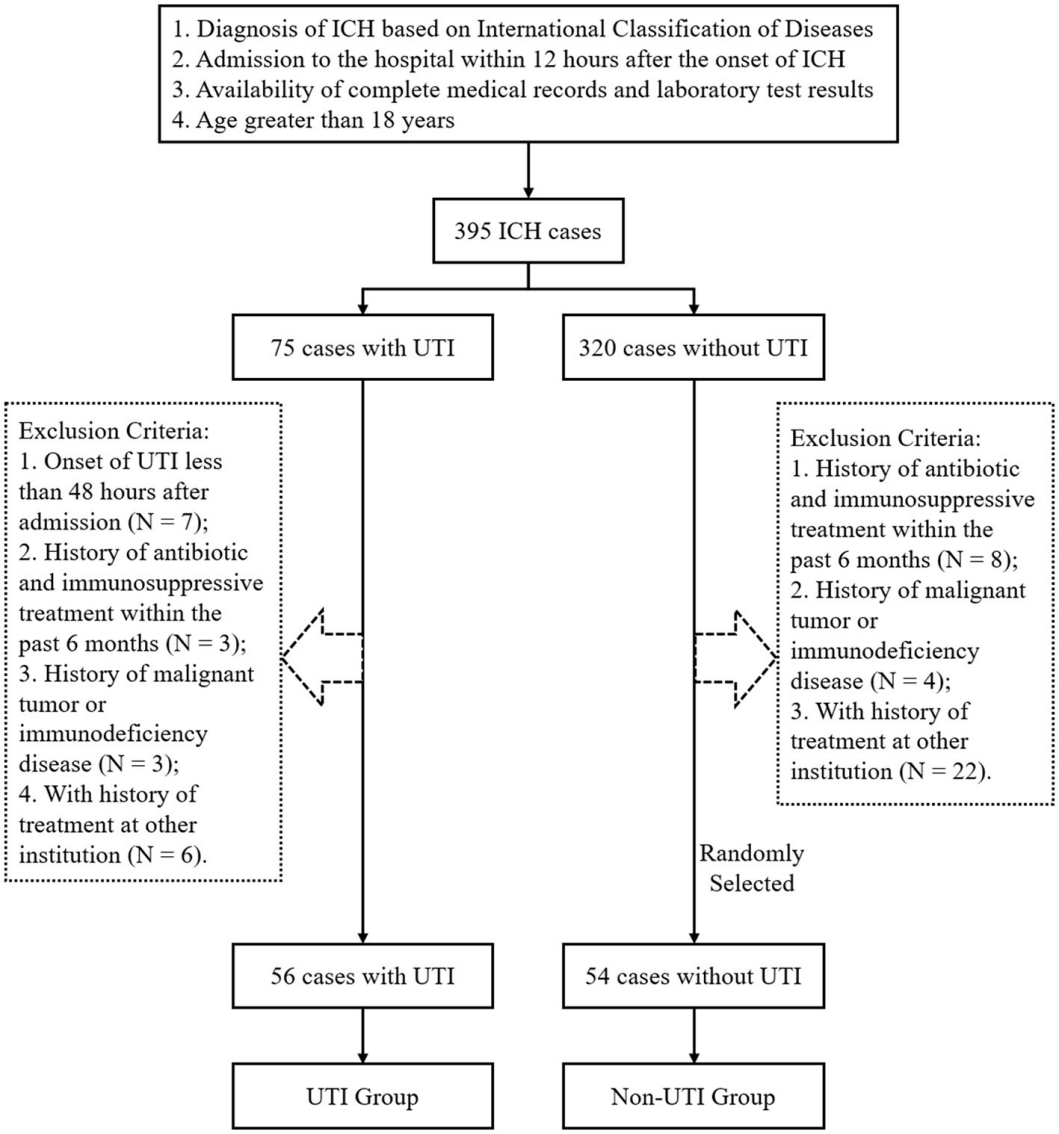
Flowchart of the study cohort selection.

In this single-center retrospective study, patients were selected from the intensive care unit of West China Hospital between January 2019 and December 2022. Traumatic or other types of intracranial hemorrhage were excluded in this research. The cohort was initially identified by reviewing the Hospital Information System (HIS) using the following criteria: 1. patients with a diagnosis of ICH (International Classification of Diseases, Tenth Revision codes, 161.x); 2. admission to the hospital within 12 h following the onset of ICH; 3. availability of complete medical records and laboratory test results; and 4. age >18 years. Among all the selected 395 ICH patients, 75 cases were diagnosed with UTI based on the European Association of Urology Urologic Infections Guidelines, cited as follows: (1) presence of any one one of the following clinical manifestations may suggest a possible UTI: new onset or worsening of fever, chills, altered mental status, malaise, lethargy with no other identifiable cause, flank pain, costovertebral angle tenderness, or acute hematuria. (2) for cases without symptoms, laboratory test results are required: A. positive results in routine urinalysis; B. bacterial growth of >10^5^ colony-forming units (CFU)/mL in a midstream voided urine specimen; and C. ≥10^3^ CFU/mL of ≥1 bacterial species in a single urine specimen for catheterized patients (30, 31). Some patients were excluded from the study for the following reasons: 1. onset of UTI <48 h after admission, suggesting they should not be classified as hospital-acquired infection (*N* = 7) (32); 2. history of antibiotic and immunosuppressive treatment within the past 6 months (*N* = 3); 3. history of malignant tumor or immunodeficiency disease (*N* = 3); 4. history of treatment at other institutions (*N* = 6). Based on these criteria, a total of 56 cases diagnosed with UTI after ICH were included in the study. Considering that skewed class proportions might negatively affect model performance, 54 non-UTI cases were randomly selected as a non-UTI cohort. The workflow of the patient selection is illustrated in Figure 1.

Clinical information was also collected, including demographic information (gender, height, weight, BMI, age, smoking, drinking, and time before admission), history (heart disease, pulmonary disease, diabetes, and hypertension), and details of treatment (surgery, intubation, corticosteroid usage, coma severity, urinary volume, and pneumonia). Coma severity was assessed using the Glasgow Coma Scale (GCS) score and classified into categorical variables, including severe brain injury (GCS score ≤ 8) and moderate brain injury (8 <GCS score ≤ 12). Urinary volume was categorized as oliguria or anuria (≤ 400 ml/day), normal urinary volume (400–2,500 ml/day), and polyuria (>2,500 ml/day).

### 2.2. Covariates

All the patients were managed with the placement of a urine drainage catheter after admission and received standard daily protocol meatal care. In our hospital, venous blood was sampled for a routine blood test and metabolic panel at admission and every 24 h in the first 3 days. Laboratory test results were chosen at two time points: Lab 1 collected at the time of admission, representing patients' condition before treatment, and Lab 2 collected at the time of 48 h after admission, representing patients' relatively stable condition after standardized therapeutic management. The following formulation was adopted when calculating the rate of change of each laboratory test results:


$$\begin{array}{l} Delta\;Lab\;\left(\Delta \;Lab\right)=\left(\frac{Lab\;2^{nd}}{Lab{\;1}^{st}}-1\right) \end{array}$$

All the collected variables are listed in Supplementary material 1.

### 2.3. Statistical analysis and feature selection

Categorical variables were described as numbers and percentages and analyzed by Pearson's chi-squared test. Continuous variables were described as value ± standard deviations or value ± quarterback range based on the results of the Shapiro–Wilk test. Two-tailed student's *t*-test or Mann–Whitney *U*-test was used for univariate analysis, as appropriate.

The clinical parameters would be selected for modeling if their *p*-values were <0.10 in the univariate analysis. Given the rather large number of laboratory covariates, the following strategies were used: first, the covariates were standardized to fit Gaussian distribution, and second, we adopted the least absolute shrinkage and selection operator (LASSO) to select the optimal parameters by using 5-fold cross-validation. Specifically, feature selection was performed on the training set in each fold, and the parameters would be determined as optimal only if they were chosen at least three times in the cross-validation.

### 2.4. Modeling strategy

To investigate which dataset should be used, four machine learning models were established by using linear discriminant analysis (LDA) algorithms with various combinations of parameters as following strategy: Model 1: a combination of clinical features and Lab 1; Model 2: a collection of clinical features, Lab 1, and Lab 2; Model 3: a combination of clinical features and ΔLab; and Model 4: a collection of clinical features, Lab 1, and ΔLab.

**Figure 2:**
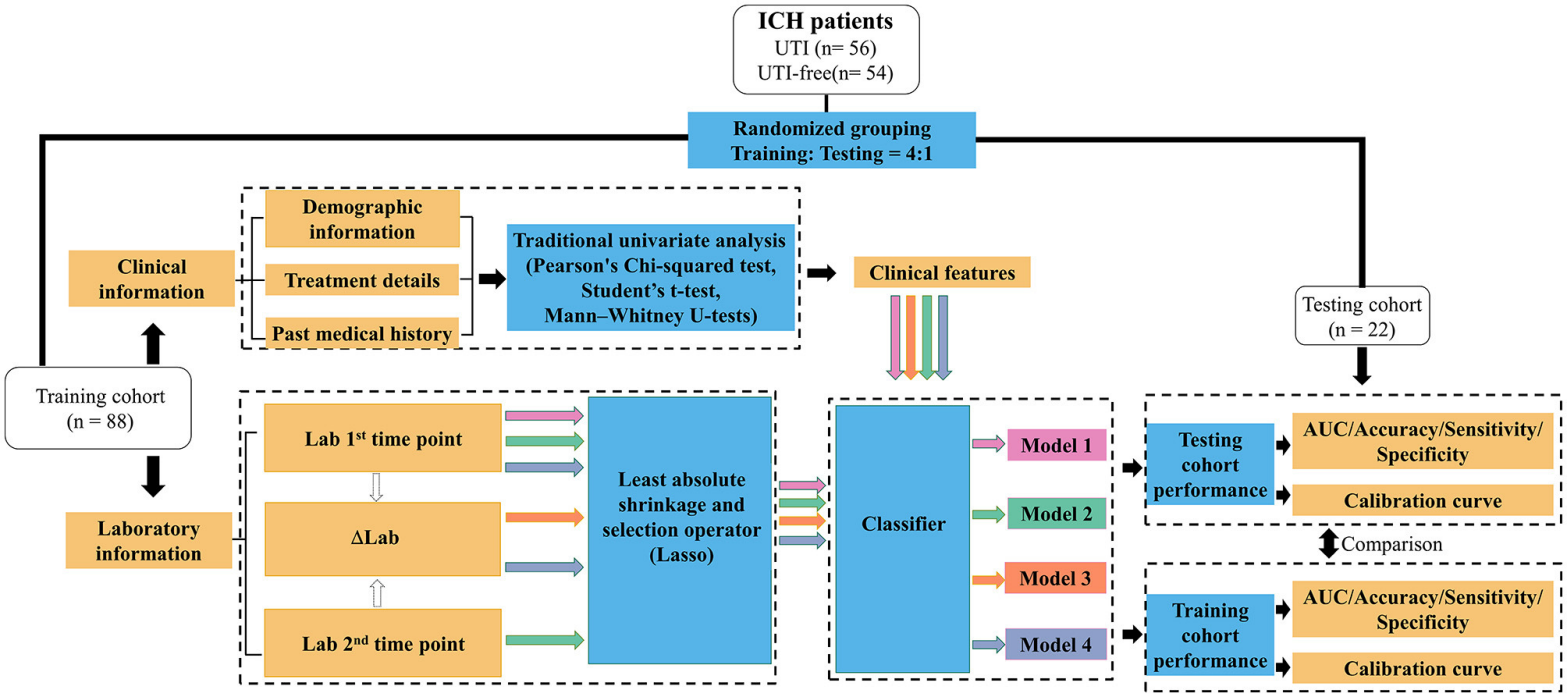
Workflow for the development and validation of machine learning models to predict the potential UTI in ICH patients. Lab 1: Laboratory results tested after patients' admission; Lab 2: Laboratory results tested during 48 h after admission; ΔLab: The rate of change of laboratory results.

Then, logistic regression, decision tree, random forest, support vector machines, and eXtreme Gradient Boosting were enrolled to further investigate if the predictive performances could be further improved using different machine learning algorithms. The machine learning models were implemented using the scikit-learn package (https://github.com/scikit-learn/scikit-learn) in Python programming language (version 3.9). Hyperparameters are set based on the recommendation of the package. Figure 2 illustrates the workflow of our model establishment and evaluation.

### 2.5. Model training and performance evaluation

The same 5-fold cross-validation as a selecting feature was used for model training and testing. Specifically, the training cohort was used to develop, optimize, and internally evaluate the performance of predictive models, while the testing cohort, which remained unseen during model training, was employed to assess testing performance. Categorical variables were converted into binary integer codes using OneHotEncoder, and continuous variables were standardized using StandardScaler (https://scikit-learn.org/stable/modules/preprocessing.html). The performance of each model was assessed using metrics such as accuracy (ACC), sensitivity, specificity, and area under the curve (AUC) values. Calibration curves were also generated to compare the predicted outcomes of the model against the observed outcomes.

## 3. Results

### 3.1. Patient clinical information

A total of 110 patients were enrolled in the study. The majority of patients in the UTI group were women (53.6%), while the non-UTI group was predominantly men (63.0%). The mean ages for the UTI and non-UTI groups were 56.4 ± 17.4 years and 51.1 ± 14.5 years, respectively. The average time before admission for the UTI group was 7.7 ± 4.6 h, compared to 6.1 ± 4.3 h for the non-UTI group. Over 70% of patients in both groups received tracheal intubation and surgery. Traditional univariate analysis revealed that corticosteroid usage (*p* < 0.001) and daily urinary volume (*p* < 0.001) were statistically significant influencing factors for UTI. Detailed clinical information and the results of the univariate analysis are listed in Table 1.

**Table 1:** Clinical information of the included patients.

	**UTI group (*n*= 56)**	**Non-UTI group (*n*= 54)**	***p*-value**
**Sex**			
Male	26 (46.4)	34 (63.0)	0.082
Female	30 (53.6)	20 (37.0)	
**Age**	56.4 ± 17.4	51.1 ± 14.5	0.084
**Height (cm)**	163.5 ± 7.5	163.8 ± 9.4	0.857
**Weight (kg)**	63.9 ± 11.2	63.8 ± 13.0	0.673
**BMI**	23.83 ± 3.36	24.0 ± 3.91	0.745
**Smoking**			
Yes	4 (7.1)	6 (11.1)	0.469
No	52 (92.9)	48 (88.9)	
**Drinking**			
Yes	2 (3.6)	4 (7.4)	0.434
No	54 (96.4)	50 (92.6)	
**Time before admission (hours)**	7.7 ± 4.6	6.1 ± 4.3	0.074
**Heart disease**			
Yes	4 (7.1)	4 (7.4)	1.000
No	52 (92.9)	50 (92.6)	
**Pulmonary disease**			
Yes	3 (5.4)	1 (1.9)	0.618
No	53 (94.6)	53 (98.1)	
**Diabetes**			
Yes	9 (16.1)	9 (16.7)	0.933
No	47 (83.9)	45 (83.3)	
**Hypertension**			
Yes	31 (55.4)	29 (53.7)	0.862
No	25 (44.6)	25 (46.3)	
**Surgery**			
Yes	43 (76.8)	45 (83.3)	0.391
No	13 (23.2)	9 (16.7)	
**Intubation**			
Yes	50 (89.3)	43 (79.6)	0.161
No	6 (10.7)	11 (20.4)	
**Corticosteroid use**			
Yes-I.V.	20 (35.7)	19 (35.2)	**<0.001**
Yes-Inhale	3 (5.4)	7 (13)	
No	33 (58.9)	28 (51.8)	
**Coma severity**			
Severe brain injury	36 (64.3)	36 (66.7)	0.793
Moderate brain injury	20 (35.7)	18 (33.3)	
**Urinary volume**			
Oliguria or anuria	3 (5.4)	1 (1.9)	**<0.001**
Normal urinary volume	11 (19.6)	18 (33.3)	
Polyuria	42 (75.0)	35 (64.8)	
**Pneumonia**			
Yes	41 (73.2)	43 (79.6)	0.429
No	15 (26.8)	11 (20.4)	

Bold values indicate that *p* < 0.05.

### 3.2. Laboratory test results at multiple time points

**Figure 3:**
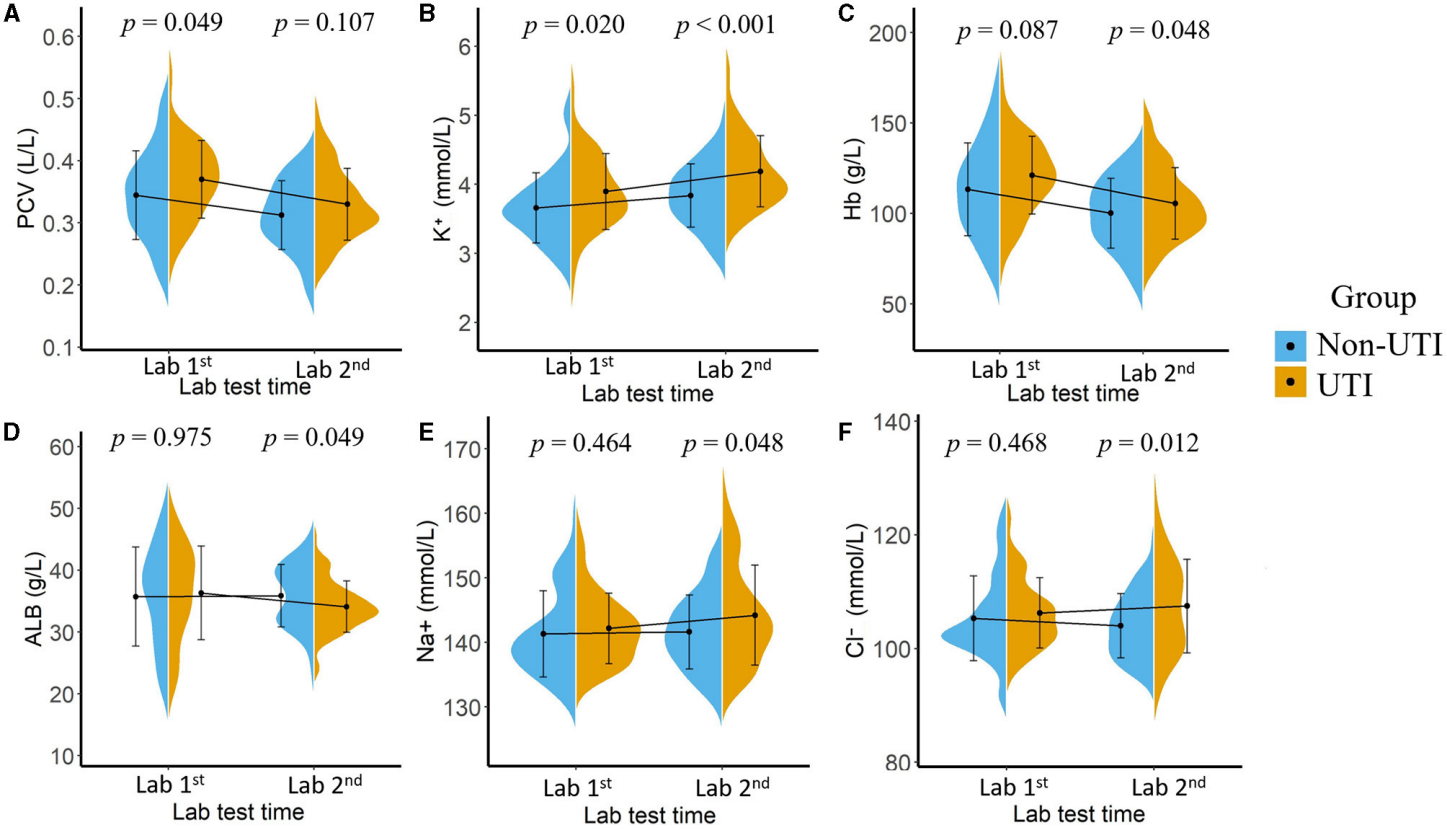
Mean value and the statistical distribution of packed cell volume, [PCV, **(A)**]; serum potassium **(B)**, hemoglobin [Hb, **(C)**], serum albumin [ALB, **(D)**], serum sodium **(E)**, and serum chloride **(F)** in the UTI group and non-UTI group. Lab 1: Laboratory results tested after patients' admission; Lab 2: Laboratory results tested 48 h after admission.

The results of our study showed there were significant differences in laboratory test results between the two groups either at the first or the second time point, as illustrated in Figure 3. For the laboratory results collected at the first time point, higher packed cell volume (PCV, *p* = 0.049) and higher serum potassium (K+, *p* = 0.020) were associated with an increased risk of UTI, whereas after receiving appropriate therapeutic intervention, laboratory tests collected at the second time point showed that higher hemoglobin (Hb, *p* = 0.048), lower albumin (ALB, *p* = 0.049), higher serum potassium (K+, *p* < 0.001), higher serum sodium (Na+, *p* = 0.048), and higher serum chloride (Cl-, *p* = 0.012) were associated with a higher risk of potential UTI.

### 3.3. Machine learning model development and validation

Based on the results of the univariate analysis, five clinical variables were selected in the subsequent model construction: gender (*p* = 0.082), age (*p* = 0.084), time before admission (*p* = 0.074), corticosteroid usage (*p* < 0.001), and urinary volume (*p* < 0.001). As for the laboratory parameters, the features selected for modeling are listed in Supplementary material 2.

**Figure 4:**
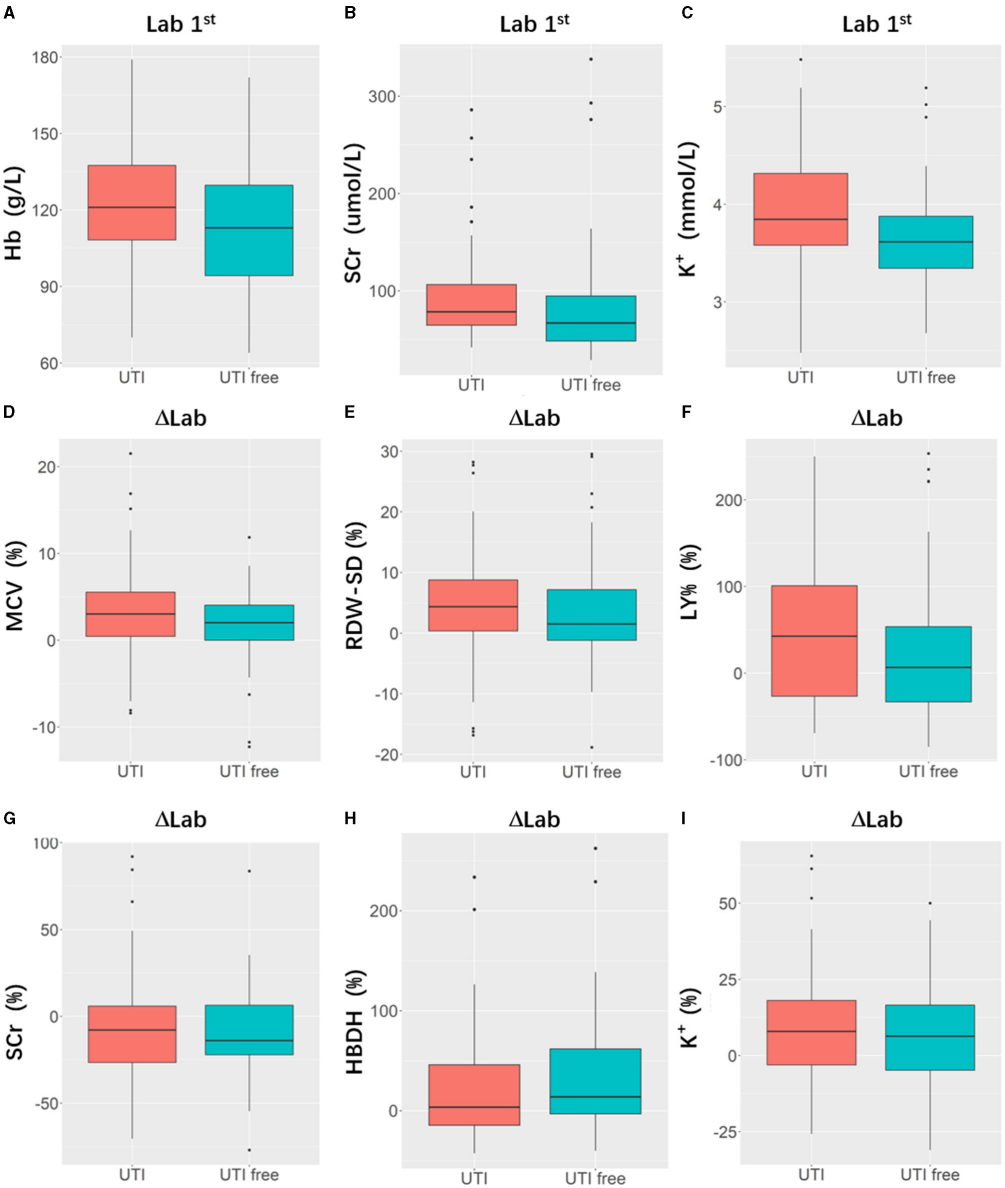
Boxplots indicating statistic distribution of the selected features for Model 4, including 3 Lab 1^st^ features (hemoglobin, [Hb, **(A)**], serum creatine, [SCr, **(B)**], serum potassium [K^+^, **(C)**]) and 6 ΔLab features (mean corpuscular volume [MCV, **(D)**], standard difference of red blood cell distribution width [RDW-SD, **(E)**], lymphocyte percentage [LY%, **(F)**], serum creatine [SCr, **(G)**], hydroxybutyrate dehydrogenase [HBDH, **(H)**], serum potassium [K^+^, **(I)**]). Lab 1: Laboratory results tested after patients' admission; ΔLab: The rate of change of laboratory results.

**Figure 5:**
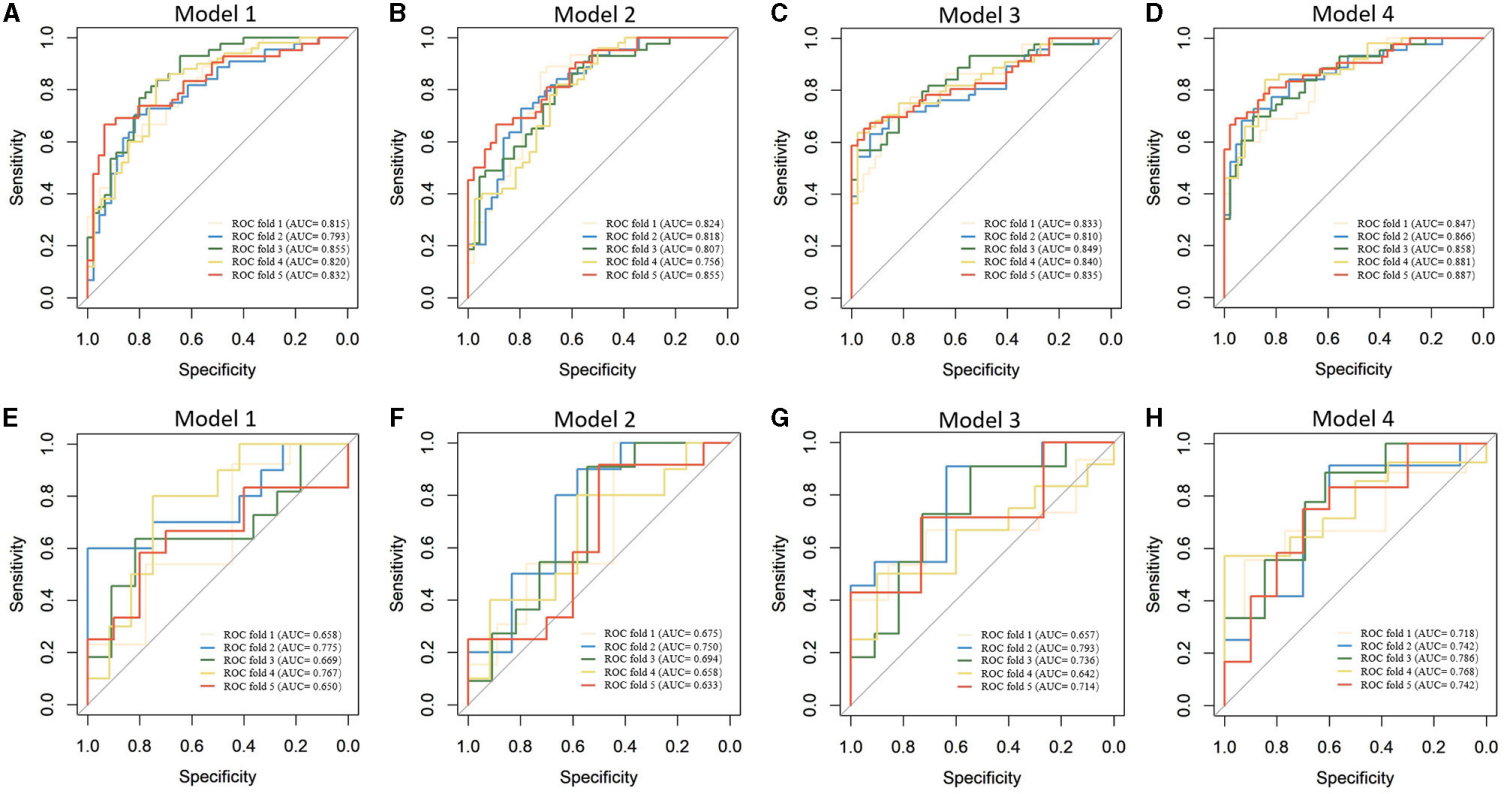
Receiver operating characteristics curves of machine learning models in predicting upcoming UTI of ICH patients. **(A–D)**. Model training; **(E–H)** Model testing.

**Figure 6:**
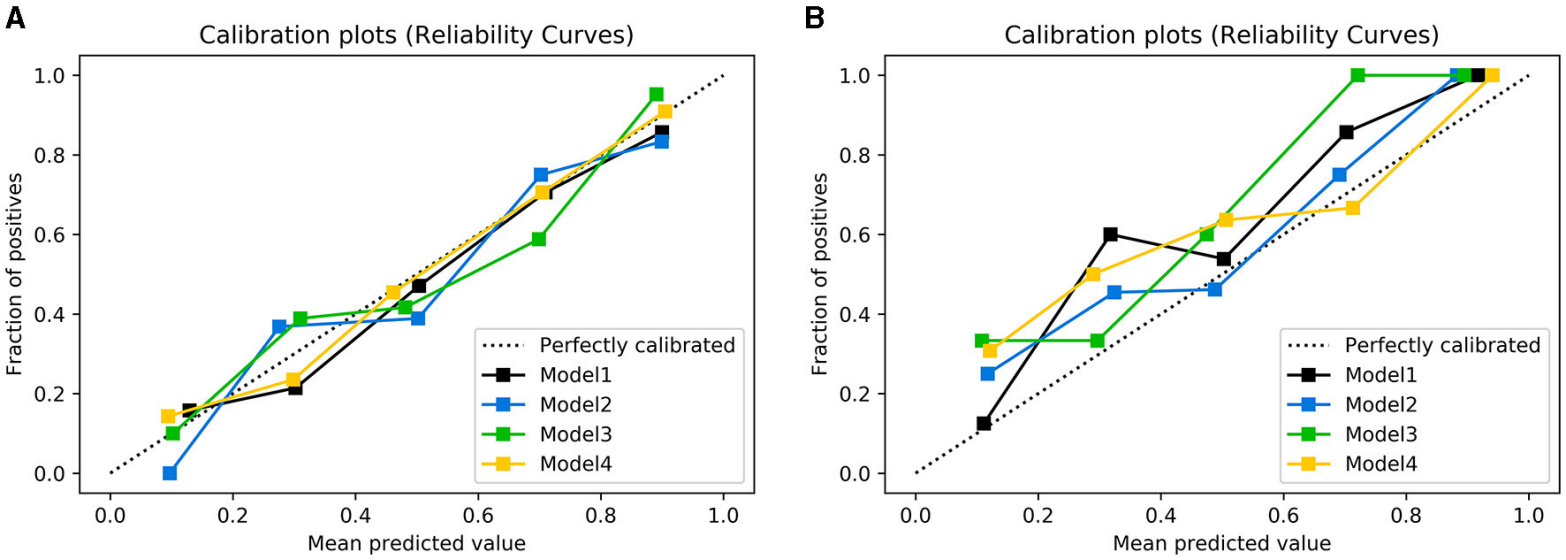
Calibration plots of observed vs. predicted UTI risk in the training cohort **(A)** and testing cohort **(B)**. The dotted line indicates a good agreement between the predicted probability of the model and the actual probability.

Table 2 presents the results of the model performance in both the training cohort and the testing cohort. Among all combinations of parameters, Model 1 achieved an ACC of 0.752 ± 0.033, sensitivity of 0.751 ± 0.034, specificity of 0.756 ± 0.044, and AUC of 0.823 ± 0.020 in model training, and 0.682 ± 0.029, 0.695 ± 0.088, 0.708 ± 0.067, and 0.704 ± 0.055 in the model test. Model 2 and Model 3 showed slight improvements in AUC (0.824 ± 0.013 and 0.833 ± 0.013 in the training cohort; 0.710 ± 0.011 and 0.708 ± 0.055 in the testing cohort). Model 4 performed the best in terms of AUC. Figure 4 shows the statistical distribution of the selected features for Model 4. More specifically, ACC, sensitivity, specificity, and AUC of model 4 in the training cohort were 0.773 ± 0.031, 0.785 ± 0.032, 0.762 ± 0.034, 0.868 ± 0.015, respectively, and in the testing cohort were 0.682 ± 0.041; 0.685 ± 0.051; 0.673 ± 0.113; 0.751 ± 0.024, respectively. An example of how LDA model 4 distinguishes the upcoming UTI group and non-UTI group in the training cohort is illustrated in Supplementary material 3. All ROC curves are summarized and illustrated in Figure 5, Supplementary material 4. The calibration curves of each model are represented in Figure 6.

**Table 2:** Machine learning model performance in predicting upcoming urinary tract infection.

**Models**		**ACC**	**Sensitivity**	**Specificity**	**AUC**
Model 1	Model training	0.752 ± 0.033	0.751 ± 0.034	0.756 ± 0.044	0.823 ± 0.020
	Model testing	0.682 ± 0.029	0.695 ± 0.088	0.708 ± 0.067	0.704 ± 0.055
Model 2	Model training	0.745 ± 0.017	0.740 ± 0.029	0.751 ± 0.009	0.824 ± 0.013
	Model testing	0.636 ± 0.076	0.624 ± 0.046	0.629 ± 0.130	0.710 ± 0.011
Model 3	Model training	0.752 ± 0.022	0.761 ± 0.026	0.743 ± 0.020	0.833 ± 0.013
	Model testing	0.664 ± 0.046	0.654 ± 0.088	0.652 ± 0.135	0.708 ± 0.055
Model 4	Model training	0.773 ± 0.031	0.785 ± 0.032	0.762 ± 0.034	0.868 ± 0.015
	Model testing	0.682 ± 0.041	0.685 ± 0.051	0.673 ± 0.113	0.751 ± 0.024

Table 3 represents the predictive performances of upcoming UTI of different machine learning models in the testing cohort. Apart from the LDA model, the decision tree model achieves an AUC of 0.636 ± 0.050, accuracy of 0.618 ± 0.084, sensitivity of 0.607 ± 0.103, and specificity of 0.618 ± 0.167. The random forest mode, support vector machine model, and eXtreme Gradient Boosting model demonstrate improved predictive performance with an AUC of 0.718 ± 0.061, 0.703 ± 0.071, and 0.687 ± 0.065, respectively. The logistic regression model achieved a relatively better performance among the recently proposed models, with an AUC of 0.728 ± 0.102, accuracy of 0.673 ± 0.045, sensitivity of 0.668 ± 0.128, and specificity of 0.681 ± 0.145. However, in summary, the LDA model achieves the best predictive performance of upcoming UTI in the testing cohort.

**Table 3:** Different machine learning model performances in predicting the upcoming UTI in the testing cohort.

**Models**	**AUC**	**Accuracy**	**Sensitivity**	**Specificity**
Linear discriminant analysis	0.751 ± 0.024	0.682 ± 0.041	0.685 ± 0.051	0.673 ± 0.113
Decision tree	0.636 ± 0.050	0.618 ± 0.084	0.607 ± 0.103	0.618 ± 0.167
Logistic regression	0.728 ± 0.102	0.673 ± 0.045	0.668 ± 0.128	0.681 ± 0.145
Random forest	0.718 ± 0.061	0.627 ± 0.053	0.671 ± 0.113	0.618 ± 0.093
Support vector machine	0.703 ± 0.071	0.673 ± 0.078	0.692 ± 0.193	0.709 ± 0.121
eXtreme Gradient Boosting	0.687 ± 0.065	0.636 ± 0.149	0.622 ± 0.155	0.671 ± 0.182

## 4. Discussion

In this retrospective cohort study, we collected clinical and multi-time-point laboratory information to construct machine learning models for predicting the potential risk of UTI in ICH patients. The models showed good performance and achieved the highest AUC of 0.868 ± 0.015 in the training cohort and 0.751 ± 0.024 in the testing cohort when multi-time-point laboratory information was used. These results suggest that our models can potentially assist in providing more effective prevention of UTI after ICH to facilitate timely intervention in clinical practice.

A large and growing body of literature has investigated the independent risk factors for UTI in stroke patients (6–9, 13–18, 33–35). With a retrospective cohort consisting of 412 cases, one study suggested that clinical and laboratory parameters collected at admission were correlated with UTI after stroke, including older age, higher serum urea, and higher serum albumin (7). Similarly, another study suggested that laboratory results collected on the 2nd day of admission were also related, including higher interleukin-6, and lower hemoglobin (14). Moreover, postvoid residual volume >100 mL, longer length of hospital stays, prolonged duration of an indwelling catheter, higher modified Rankin scale score, higher white blood cell count, higher copeptin, and higher procalcitonin were also reported to be high-risk factors for UTI after stroke (6, 8, 9, 13, 15–18, 33–35). Our analysis identified several features that were associated with a higher risk of UTI in ICH patients. Due to the large number of variables collected at the clinical practice, it is also reasonable to assume that the relationship between a single variable and the risk of UTI after stroke is still inconsistent, as suggested by these results, and traditional statistical analysis may perform below expectations.

Compared with the traditional multivariate logistic model, the scalability of machine learning algorithms allows a systematic analysis of a large dataset with numerous variables. One study suggested that the machine learning model showed good performance in predicting the UTI with an AUC of 0.808 and ACC of 0.703, with the selected parameters based on recommendations and a review of literature (Supplementary material 5) (29). Model 1 in our research was designed with the same strategy. Although the sample size of our research is rather small, this model achieved a similar performance (model training, AUC = 0.823; model test, AUC= 0.704) to the previous one. With the fast progression of ICH, the prediction of upcoming UTIs should rely on multi-time-point data, and variation of the parameters should also be considered. Therefore, in the study, we managed to adapt machine learning algorithms to enroll the most relevant features from a multi-time-point dataset.

The modeling strategy of Models 2, 3, and 4 in our research was recommended by experienced neuro-intensive care physicians. Model 2 was established based on the multi-time-point parameters and showed a similar performance to Model 1 with an AUC of 0.824 in the training cohort and 0.710 in the testing cohort. This finding may be attributed to traditional machine learning models taking enrolled features as independent factors rather than utilizing their internal relationships (36). Additionally, the delta model (Model 3), which enrolled the rate of change of laboratory parameters, exhibited similar predictive performance to Model 1 (training cohort, AUC = 0.833; testing cohort, AUC= 0.708), indicating that the rate of change may play an equally important role as the values of predictive indicators. However, Model 4, which was designed to simulate clinical decision-making, outperformed all other models with an increase in AUC in both training and testing cohorts (training cohort, AUC = 0.868; testing cohort, AUC= 0.751). On the one hand, our research provided a model for predicting UTIs to assist in therapeutic decision-making. By enabling early detection, personalized care plans, optimized resource allocation, reduced antibiotic overuse, and improved patient outcomes, the model empowers healthcare teams to intervene proactively and tailor treatments for individual patients, ultimately enhancing patient care, reducing complications, and fostering a data-driven approach to medicine while maintaining the vital role of clinical expertise in decision-making. On the other hand, these results also highlighted the importance of considering the variation of laboratory results in conjunction with the patient's clinical presentation as a single laboratory finding may not be enough to diagnose a UTI accurately. Monitoring these laboratory findings over time can provide necessary information about the progression of disease and the effectiveness of treatment, helping healthcare providers optimize patient care to improve outcomes.

It should be noted that clinical factors such as the indwelling time of the urinary catheter (37) and length of hospital stay (29) have been related to the increased risk of UTI in previous research studies. Compared with their study, the primary purpose of this research was to identify patients who are at an increased risk of developing UTI after ICH at an early stage. Hence, all the incorporated features were gathered within a 3-day window after admission, with features requiring a collection period exceeding 3 days, such as indwelling time, being excluded from consideration. It is also worth noting that among all the combinations of features, gender and serum creatinine levels were assigned with the highest average coefficients in Model 4 (Supplementary material 6). Previous studies have recognized female sex as a common clinical risk factor due to anatomical differences, where the shorter distance between the urethral and anal opening and vaginal cavity may increase the risk of infection (3). Serum creatinine (Scr) is often related to kidney function in clinical practice, where a blocked urinary tract or chronic infection of the kidney may increase the level and potentially cause an upcoming UTI. Several retrospective cohort studies also identified significant differences in SCr between UTI and non-UTI patients (38, 39). In summary, these results further support the predictive performance of our model and extend its clinical interpretability.

We also found that there is a significant relationship between the ACC of Model 4 and hospitalization time. Given that the laboratory information used in modeling was collected in the early stages, it is clinically relevant to investigate if our model can still be effective in predicting long-term UTI occurrences. We summarized and analyzed the ACC values of UTI cases in the 5-fold test group and found that the model performed much better in predicting UTI onset within 14 days (31 cases, ACC = 0.806) compared to 14 days or more (25 cases, ACC = 0.560). This discrepancy can be attributed to the fact that the features used in this study primarily reflect the patient's early condition after ICH. However, as the disease may progress fast, clinical treatment intervention and patient management play a more critical role in determining whether a UTI will occur. Therefore, we must acknowledge that our model is better suited for predicting UTIs occurring within 2 weeks.

This study has several potential limitations. First, its retrospective cohort design may introduce inherent selection bias. Second, the relatively small sample size may limit the interpretability of the identified features, necessitating a larger-scale study to corroborate our findings. Third, given that this was a single-center study, data from multiple centers are needed to validate the model's performance. A multi-center study with a larger sample size is essential to verify our results.

## 5. Conclusion

In this study, machine learning-based predictive models were developed and validated by using statistics collected at multiple time points for UTI prediction in ICH cases from a neuro-intensive care unit. The model showed favorable performance and clinical interpretability but should be verified in large, multi-center research in future.

## Data availability statement

The raw datasets supporting the conclusions of this article will be made available by the authors on request.

## Author contributions

Conceptualization: WC and JX. Methodology and writing—original draft preparation: YZ and CC. Validation: ZH, HW, and XT. Formal analysis and writing—review and editing: CC. Investigation: JY. Resources and data curation: YZ. Funding acquisition: JX. All authors contributed to the article and approved the submitted version.
