# Supplementary material for: Prediction of upcoming urinary tract infection after intracerebral hemorrhage: a machine learning approach based on statistics collected at multiple time points
Source: Front Neurol. 2023 Sep 14;14:1223680. doi: 10.3389/fneur.2023.1223680 (PMC10538571; doi:10.3389/fneur.2023.1223680)
Supplement: Supplementary file 1 [file Table_1.DOCX]

|  |  | Covariates | | | | |
| --- | --- | --- | --- | --- | --- | --- |
| Clinical information | Demographic information | Gender | Age | Height | Weight | Time before admission |
|  |  | BMI | Smoking | Drinking |  |  |
|  |  | Heart disease | Pulmonary disease | Diabetes | Hypertension |  |
|  | Therapeutic information | Surgery | Intubation | Corticosteroid usage | Coma severity | Urinary volume |
|  |  | Pneumonia |  |  |  |  |
|  |  |  |  |  |  |  |
| Laboratory information | Laboratory results  (Lab 1^st^, Lab 2^nd^ and ΔLab) | RBC | Hb | PCV | MCV | MCH |
|  |  | MCHC | RDW-CV | RDW-SD | PLT | WBC |
|  |  | GR% | LY% | MoNo% | GR | LY |
|  |  | MoNo | TBIL | DBIL | IDBL | TBA |
|  |  | ALT | AST | AST/ALT | ALP | GGT |
|  |  | TP | ALB | GLB | ALB/GLB | GLU |
|  |  | BUN | Cr | eGFR | Cys C | UA |
|  |  | TG | CHOL | CK | LDH | HBDH |
|  |  | Na^+^ | K^+^ | Cl^-^ | CO2CP |  |

**Supplementary Material 1** Summary of collected clinical information and laboratory information

Lab 1st: Laboratory results tested after patients’ admission

Lab 2nd: Laboratory results tested during 48-72 hours after admission

ΔLab: The rate of change of laboratory results

RBC: red Blood Cell Count

Hb: hemoglobin

PCV: packed cell volume

MCV: mean corpuscular volume

MCH: mean corpuscular hemoglobin

MCHC: mean corpuscular hemoglobin concentration

RDW-CV: coefficient of Variation of red blood cell distribution width

RDW-SD: standard difference of red blood cell distribution width

PLT: platelet count

WBC: white blood cell count

GR%: granulocyte percentage

LY%: lymphocyte percentage

MoNo%: monocyte percentage

GR: granulocyte

LY: lymphocyte

MoNo: monocyte

TBIL: total bilirubin

DBIL: direct bilirubin

IDBL: indirect bilirubin

TBA: total bile acid

ALT: alanine aminotransferase

AST: aspartate aminotransferase

ALP: alkaline phosphatase

GGT: glutamyl transpeptidase

TP: total Protein

ALB: albumin

GLB: globulin

GLU: glucose

BUN: urea

Cr: creatinine

eGFR: estimated glomerular filtration rate

Cys C: serous levels of cystatin C

UA: uric acid

TG: triglyceride

CHOL: cholesterol

CK: creatine kinase

LDH: lactate dehydrogenase

HBDH: hydroxybutyrate dehydrogenase

Na+: serum sodium

K+: serum potassium

Cl-: serum chloride

CO2CP: carbon dioxide-combining power
