# Supplementary material for: Prediction of upcoming urinary tract infection after intracerebral hemorrhage: a machine learning approach based on statistics collected at multiple time points
Source: Front Neurol. 2023 Sep 14;14:1223680. doi: 10.3389/fneur.2023.1223680 (PMC10538571; doi:10.3389/fneur.2023.1223680)
Supplement: Supplementary file 2 [file Table_2.DOCX]

**Supplementary Material 2** Selected laboratory features in predictive models

| Models | Enrolled laboratory features | | | | | | | | |
| --- | --- | --- | --- | --- | --- | --- | --- | --- | --- |
|  | Lab 1^st^ | | | Lab 2^nd^ | | | ΔLab | | |
| Model 1 | RBC | Hb | MCHC |  |  |  |  |  |  |
|  | RDW-CV | EOS | ALPA |  |  |  |  |  |  |
|  | SCr | TG | K^+^ |  |  |  |  |  |  |
| Model 2 | Hb | EOS | TG | RDW-SD | GR% | LY% |  |  |  |
|  | K^+^ |  |  | TBA | HDBD |  |  |  |  |
| Model 3 |  |  |  |  |  |  | RBC | MCV | RDW-SD |
|  |  |  |  |  |  |  | LY% | ALB/GLB | SCr |
|  |  |  |  |  |  |  | eGFR | HBDH | K+ |
| Model 4 | RBC | SCr | K^+^ |  |  |  | MCV | RDW-SD | LY% |
|  |  |  |  |  |  |  | SCr | HBDH | K+ |

Lab 1^st^: Laboratory results tested after patients’ admission

Lab 2^nd^: Laboratory results tested during 48-72 hours after admission

ΔLab: The rate of change of laboratory results

RBC: red Blood Cell Count

Hb: hemoglobin

MCV: mean corpuscular volume

MCHC: mean corpuscular hemoglobin concentration

RDW-CV: red blood cell distribution width

RDW-SD: red blood cell distribution width

EOS: eosinophil

GR%: granulocyte percentage

LY%: lymphocyte percentage

TBA: total bile acid

ALP: alkaline phosphatase

ALB: albumin

GLB: globulin

SCr: serum creatinine

eGFR: estimated glomerular filtration rate

TG: triglyceride

HBDH: hydroxybutyrate dehydrogenase

K+: serum potassium
