# Supplementary material for: Prediction of upcoming urinary tract infection after intracerebral hemorrhage: a machine learning approach based on statistics collected at multiple time points
Source: Front Neurol. 2023 Sep 14;14:1223680. doi: 10.3389/fneur.2023.1223680 (PMC10538571; doi:10.3389/fneur.2023.1223680)

**Supplementary Material 3** An example of the model training process in the training cohort. Distribution of the direct LDA score determining non-UTI (A) and UTI group (B) illustrates a promising performance of the Lasso (clinical features, Lab 1^st^, and ΔLab ) + LDA algorithms.


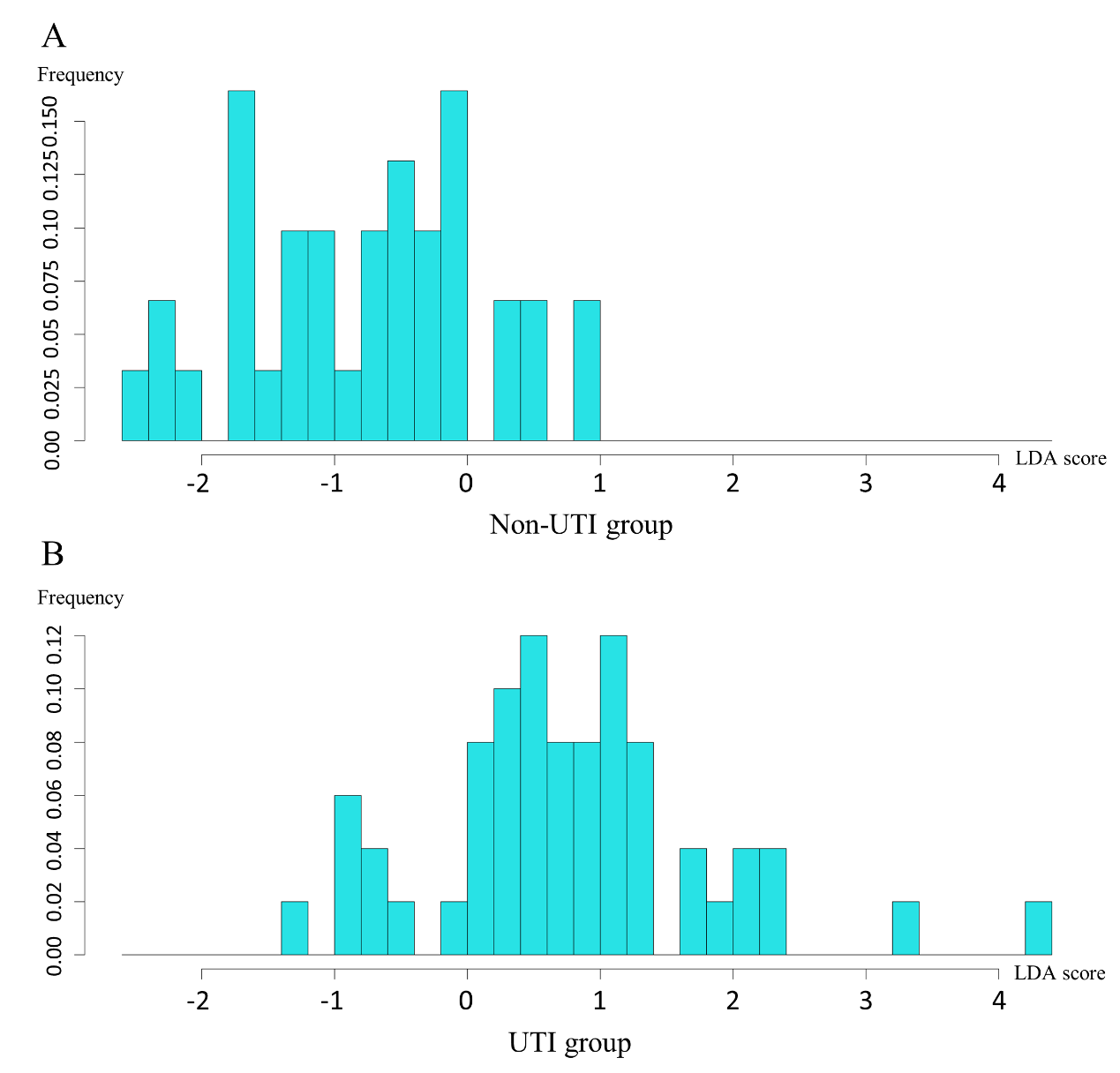

Supplement: Supplementary file 3 [file Table_3.DOCX]
