# Supplementary material for: Prediction of upcoming urinary tract infection after intracerebral hemorrhage: a machine learning approach based on statistics collected at multiple time points
Source: Front Neurol. 2023 Sep 14;14:1223680. doi: 10.3389/fneur.2023.1223680 (PMC10538571; doi:10.3389/fneur.2023.1223680)

**Supplementary material 4** Average receiver operating characteristics curves of machine learning models in predicting upcoming UTI of ICH patients in training cohort (A) and testing cohort (B).


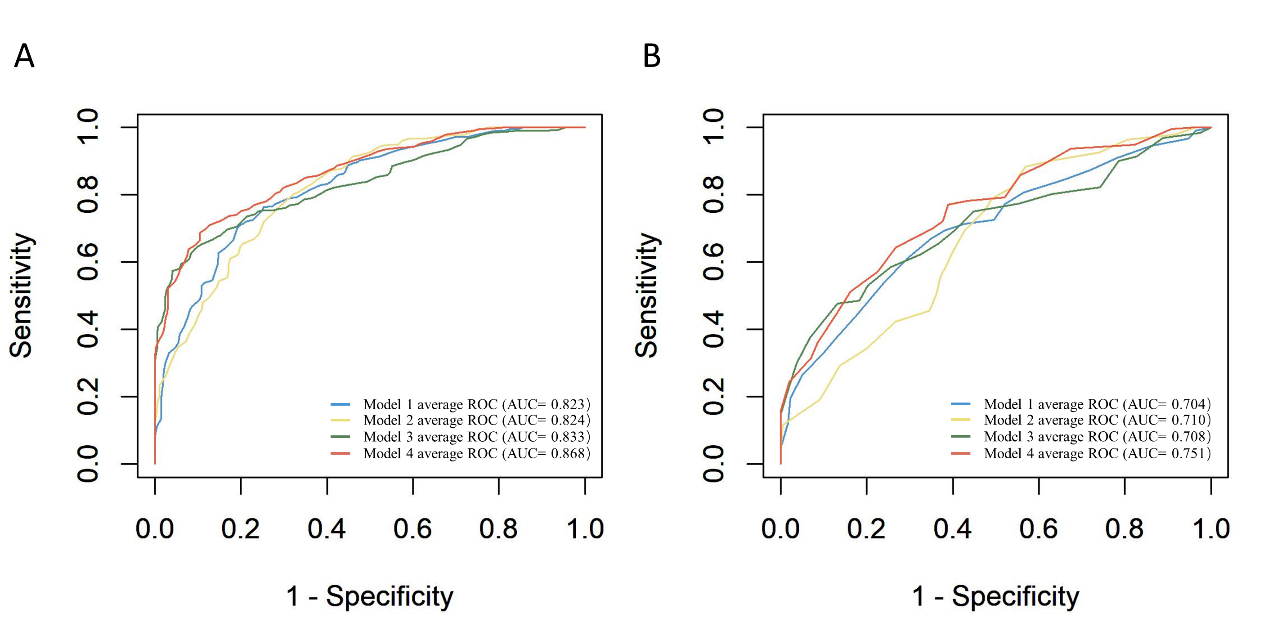

Supplement: Supplementary file 4 [file Table_4.DOCX]
