# Supplementary material for: Prediction of upcoming urinary tract infection after intracerebral hemorrhage: a machine learning approach based on statistics collected at multiple time points
Source: Front Neurol. 2023 Sep 14;14:1223680. doi: 10.3389/fneur.2023.1223680 (PMC10538571; doi:10.3389/fneur.2023.1223680)
Supplement: Supplementary file 5 [file Table_5.DOCX]

**Supplementary material 5** Comparison of UTI predictive performances in testing cohort across different studies

| Classifier | Features | Enrolled patients | AUC | Accuracy |
| --- | --- | --- | --- | --- |
| LDA | Clinical information,  Laboratory tests,  Δ Laboratory tests | ICH patients | 0.751 | 0.682 |
| ELM [26] | Clinical information | Post-stroke immobile patients | 0.808 | 0.703 |
| DT* [33] | Clinical information | NICU patients | 0.920 | 0.966 |

*Predictive performances in the training cohort.
