# Supplementary material for: Prediction of upcoming urinary tract infection after intracerebral hemorrhage: a machine learning approach based on statistics collected at multiple time points
Source: Front Neurol. 2023 Sep 14;14:1223680. doi: 10.3389/fneur.2023.1223680 (PMC10538571; doi:10.3389/fneur.2023.1223680)
Supplement: Supplementary file 6 [file Table_6.DOCX]

**Supplementary material 6** Average weights of the enrolled feature of LDA model (clinical features, Lab 1^st^, and ΔLab ) in the five-fold validation tests


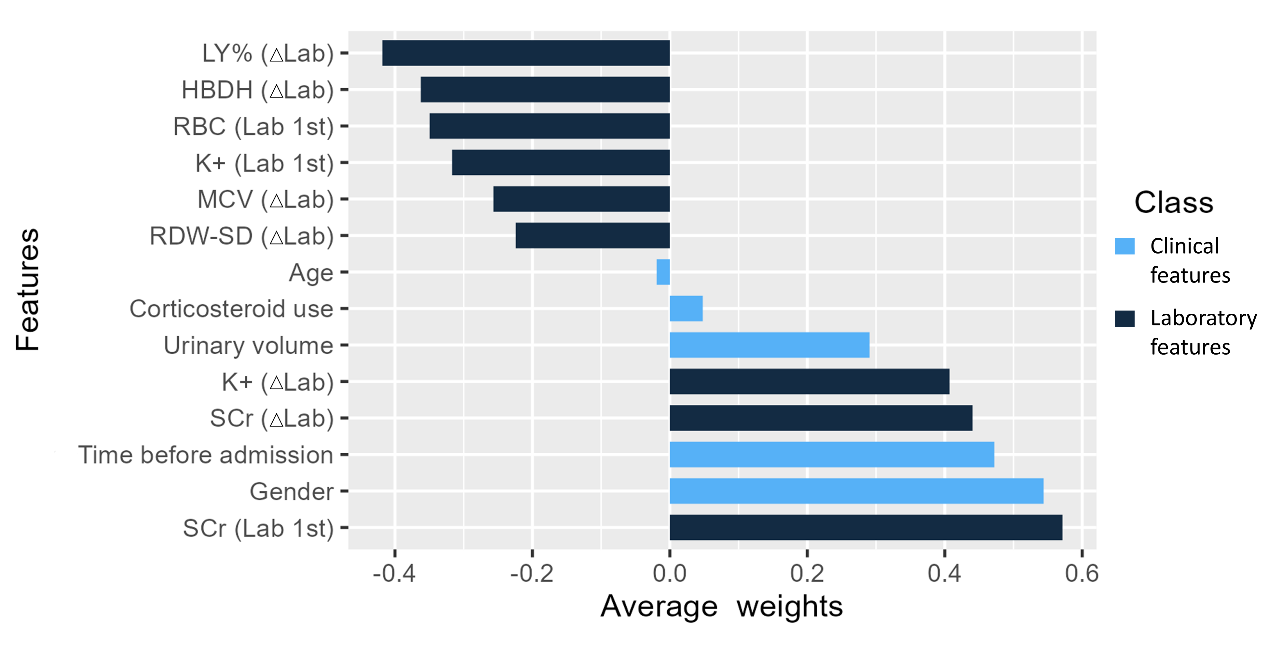


Lab 1^st^: Laboratory results tested after patients’ admission

△Lab: The rate of change of laboratory results
